# Association of VEGF With Antianhedonic Effects of Repeated-Dose Intravenous Ketamine in Treatment-Refractory Depression

**DOI:** 10.3389/fpsyt.2021.780975

**Published:** 2021-12-03

**Authors:** Wei Zheng, Li-Mei Gu, Yan-Ling Zhou, Cheng-Yu Wang, Xiao-Feng Lan, Bin Zhang, Hai-Shan Shi, Dan-Feng Wang, Yu-Ping Ning

**Affiliations:** ^1^The Affiliated Brain Hospital of Guangzhou Medical University (Guangzhou Huiai Hospital), Guangzhou, China; ^2^The First School of Clinical Medicine, Southern Medical University, Guangzhou, China

**Keywords:** ketamine, VEGF, antianhedonic effect, major depressive disorder, response

## Abstract

**Objectives:** To first explore the role of plasma vascular endothelial growth factor (VEGF) concentrations in ketamine's antianhedonic effects, focusing on Chinese patients with treatment-refractory depression (TRD).

**Methods:** Seventy-eight patients with treatment-refractory major depressive disorder (MDD) or bipolar disorder (BD) were treated with six ketamine infusions (0.5 mg/kg). Levels of anhedonia were measured using the Montgomery–Åsberg Depression Rating Scale (MADRS) anhedonia item at baseline, day 13 and 26. Plasma VEGF concentrations were examined at the same time points as the MADRS.

**Results:** Despite a significant reduction in anhedonia symptoms in individuals with treatment-refractory MDD (*n* = 59) or BD (*n* = 19) after they received repeated-dose ketamine infusions (*p* < 0.05), no significant changes in plasma VEGF concentrations were found at day 13 when compared to baseline (*p* > 0.05). The alteration of plasma VEGF concentrations did not differ between antianhedonic responders and non-responders at days 13 and 26 (all *p*s > 0.05). Additionally, no significant correlations were observed between the antianhedonic response to ketamine and plasma VEGF concentrations (all *p*s > 0.05).

**Conclusion:** This preliminary study suggests that the antianhedonic effects of ketamine are not mediated by VEGF.

## Introduction

Anhedonia, a reduced capacity for pleasure, is regarded as one of the typical characteristics of major depressive disorder (MDD) and bipolar depression (BD) (1) and appears to occur irrespective of other depressive symptoms (2, 3). Anhedonia is a robust predictor of poor outcomes (4) and suicidal ideation independent of neurocognitive dysfunction and affective symptoms (5), suggesting that it appears to be an independent somatic domain in mood disorders (3). As a residual interepisodic symptom, anhedonia has been commonly described in patients suffering from treatment-refractory depression (TRD) treated with conventional pharmacotherapy (6). Patients with mood disorders, especially those with TRD, frequently endorse disturbance in reward capacity, providing the impetus for exploring novel agents and treatment approaches (7, 8).

Ketamine, as a dissociative anesthetic, is currently evaluated as a rapid-acting antidepressant. In addition to the rapid effect on depressive symptoms, ketamine also has rapid and robust effects on anhedonia symptoms (1, 9, 10) and suicidal ideation (11–13) in treatment-refractory BD and MDD. When compared with placebo, a single ketamine infusion could rapidly ameliorate anhedonia symptoms in individuals suffering from treatment-refractory BD; the reduction in anhedonia symptoms occurred within 40 min and lasted up to 14 days (10). Interestingly, ketamine's antianhedonic effects occur independently of the reduction in depressive symptoms (10).

Accumulating evidence has implicated neurotrophic factors including brain-derived neurotrophic factor (BDNF) (14–16) and vascular endothelial growth factor (VEGF) (15–17) in the MDD and BD pathophysiology. VEGF can potentially mediate the antidepressant effects of ketamine (18, 19) and typical antidepressants (20). Similarly, serum BDNF levels were increased in chronic ketamine users (21) and change in plasma BDNF levels following subanesthetic ketamine infusion are associated with acute and 24 h resting-state functional connectivity (RSFC) changes (22). Findings on the association of VEGF and ketamine's antidepressant effects have been inconsistent (18, 23–25). For example, the expression of VEGF is necessary for the antidepressant-like behaviors of ketamine (18, 19). A recent study supported a role for VEGF in the antidepressant action of ketamine (25), but two recent studies found that ketamine does not change the plasma concentrations of VEGF (23, 24). However, evidence on the role of plasma VEGF concentrations in ketamine's antianhedonic effects is still lacking.

Therefore, the main aim of this current study, which employed a real-world design, is to determine the role of plasma VEGF concentrations in the antianhedonic effects of repeated-dose intravenous ketamine (0.5 mg/kg) administered thrice weekly over 2 weeks, focusing on Chinese subjects experiencing treatment-refractory MDD or BD.

## Methods

### Study Design and Population

Data were collected from an open-label, real-world ketamine clinical trial (registration number: ChiCTR-OOC-17012239). IRB approval of the Affiliated Brain Hospital of Guangzhou Medical University was obtained for this study (Ethical Application Ref: 2016030). All participants gave written informed consent. In this study, we specifically report the relationship of plasma VEGF concentrations and antianhedonic effects of subanaesthetic doses of ketamine, focusing on individuals suffering from treatment-refractory MDD or BD. The detailed study design, study population and clinical findings of this single-center open-label ketamine clinical study were described in our early studies (26, 27). Briefly, seventy-eight subjects aged between 18 and 65 years were recruited, with a diagnosis of major depressive episode (MED)–MDD or BD–using DSM-5 criteria. In this study, each patient was required to score ≥ 17 points on the 17-item Hamilton Depression Rating Scale (HAMD) (28, 29), experiencing TRD defined as failure to respond to at least two pharmacological therapies for the current MDE (30). Patients with other psychiatric disorders such as drug/alcohol dependence or schizophrenia were excluded, but a comorbidity of obsessive compulsive disorder (OCD) or anxiety disorder was permitted if it was not judged to be the primary presenting problem. Similar to prior studies (26, 27), each participant received six ketamine infusions (0.5 mg/kg over 40 min).

### Antianhedonic Response

Similar to several early studies (31, 32), the Montgomery–Åsberg Depression Rating Scale (MADRS) anhedonia item including items 1 (apparent sadness), 2 (reported sadness), 6 (concentration difficulties), 7 (lassitude), and 8 (inability to feel) was also used in this study to assess anhedonia symptoms at baseline, day 13 and 26 (at the 1 day and 2 week follow-ups after completing the last infusion, respectively). Antianhedonic response was defined as at least a 50% reduction in MADRS anhedonia item scores on day 13.

### Measurement of Plasma VEGF Concentrations

All blood samples of seventy-eight subjects with treatment-refractory MDD or BD were collected preinfusion and again at days 13 and 26. Consistent with a recent study (24), a Human VEGF Immunoassay enzyme-linked immunosorbent assay (ELISA) kit (R&D Systems, Minneapolis, USA) was used to measure the plasma concentrations of VEGF.

### Statistical Analysis

All statistical analyses were conducted using SPSS 24.0 statistical software focusing on Chinese patients suffering from treatment-refractory MDD or BD, with a significance level of 0.05 (two-sided). We performed a two-sample *t*-test and/or a Mann–Whitney U test as well as a chi-square test and/or a Fisher's exact test to compare the differences in baseline plasma concentrations of VEGF and demographic and clinical features between the two groups (patients with and without antianhedonic response), if necessary. A linear mixed model was conducted for changes in anhedonia symptoms as measured by MADRS and the plasma concentrations of VEGF over time between the two groups, with Bonferroni correction for the time points examined. Correlation analyses were conducted to determine the relationship of the effects of ketamine on anhedonia symptoms and the plasma concentrations of VEGF.

## Results

Table 1 presents the demographic and clinical data of the patients suffering from treatment-refractory MDD (*n* = 59) or BD (*n* = 19) who received repeated ketamine infusions and provided a blood sample at baseline. Antianhedonic non-responders had a significantly higher history of psychiatric hospitalization than antianhedonic responders (*p* < 0.05).

**Table 1:** Demographic and clinical characteristics of subjects suffering from TRD.

**Variables**	**Total sample** **(*****n*** **= 78)**		**Antianhedonic responders** **(*****n*** **= 38)**		**Antianhedonic non-responders** **(*****n*** **= 40)**		**Statistics**		
	***N***	**%**	***N***	**%**	***N***	**%**	**χ^2^**	**df**	***p-*value**
Male	39	50.0	20	52.6	19	47.5	0.2	1	0.65
Married	39	50.0	21	55.3	18	45.0	0.8	1	0.34
Employed	29	37.2	17	44.7	12	30.0	1.8	1	0.18
No history of psychiatric hospitalization	53	67.9	31	81.6	22	55.0	6.3	1	**0.01**
Having a family history of psychiatric disorders	32	41.0	13	34.2	19	47.5	1.4	1	0.23
On ADs two or more	10	12.8	4	10.5	6	15.0	0.3	1	0.56
On APs	46	59.0	21	55.3	25	62.5	0.4	1	0.52
On mood stabilizers	24	30.8	10	26.3	14	35.0	0.7	1	0.41
On benzodiazepines	31	39.7	14	36.8	17	42.5	0.3	1	0.61
On anxiolytics	36	46.2	18	47.4	18	45.0	0.04	1	0.83
On anticholinergics	12	15.4	6	15.8	6	15.0	0.01	1	0.92
Current smoking	18	23.1	9	23.7	9	22.5	0.02	1	0.90
Current drinking	4	5.1	1	2.6	3	7.5	—^a^	—^a^	0.33
	**Mean**	**SD**	**Mean**	**SD**	**Mean**	**SD**	**T/Z**	**df**	***p-*** **value**
Age (years)	34.6	11.8	35.1	11.5	34.1	12.2	0.4	76	0.70
Education (years)	12.3	3.5	12.0	3.3	12.5	3.6	−0.6	76	0.55
BMI (kg/m^2^)	22.7	3.4	23.3	3.7	22.1	3.1	1.6	76	0.12
Age of onset (years)	25.8	11.4	27.2	12.3	24.4	10.5	1.1	76	0.29
Duration of illness (months)	109.5	104.2	98.8	104.6	119.6	104.3	−1.0	—^b^	0.31
FLUeq (mg/day)	36.7	23.0	38.5	24.1	35.1	22.2	−0.6	—^b^	0.53
CPZeq (mg/day)	172.3	125.6	144.9	102.6	195.3	139.9	−1.4	—^b^	0.16
Baseline MADRS total scores	31.5	7.4	31.0	7.0	31.9	7.8	−0.5	76	0.62
Baseline MADRS anhedonia item scores	19.9	4.6	19.5	4.6	20.3	4.7	−0.8	76	0.45
Baseline plasma VEGF concentrations (ng/ml)	30.7	48.2	34.0	47.2	27.5	49.5	−0.7	—^b^	0.46

a *Fisher's exact test*;

b *Mann-Whitney U test. Bolded values are p < 0.05. Ads, Antidepressants; Aps, antipsychotics; BMI, body mass index; CPZeq, chlorpromazine equivalent milligrams; FLUeq, Fluoxetine equivalents equals; MADRS, Montgomery–Åsberg Depression Rating Scale; VEGF, vascular endothelial growth factor; TRD, treatment-refractory depression*.

**Figure 1:**
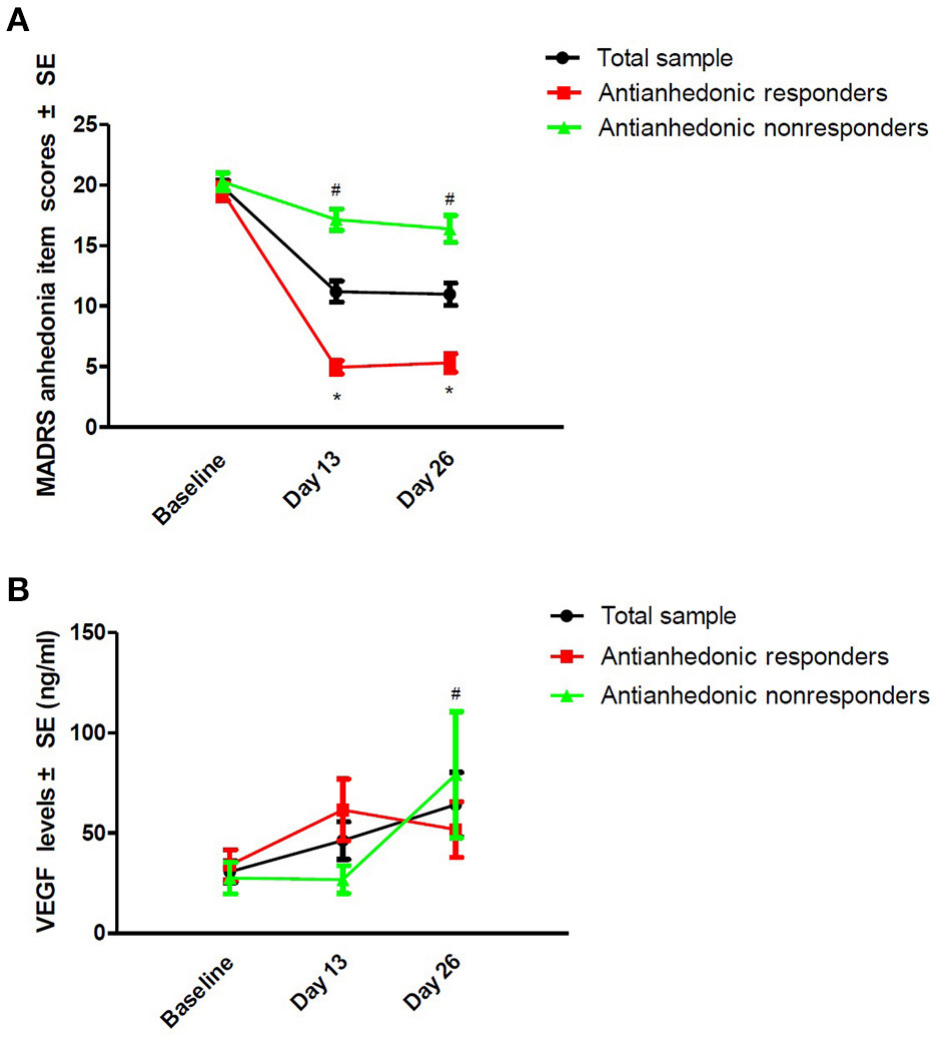
Change in anhedonia symptoms **(A)** and plasma VEGF concentrations **(B)** in subjects suffering from TRD. ^#^Significant difference was found when compared to baseline at the indicated times (*p* < 0.05). *Significant difference was found between antianhedonic responders and non-responders at the indicated times (*p* < 0.05). MADRS, the Montgomery-Asberg Depression Rating Scale; SE, standard error; TRD, treatment-refractory depression; VEGF, vascular endothelial growth factor.

Thirty-eight patients (48.7%, 95% Cl = 37.4–60.1%) fulfilled the criteria for antianhedonic response. As depicted in Table 2, significant time main effects were found regarding MADRS anhedonia item scores and plasma VEGF concentrations (all *ps* < 0.05). No significant group main effects or group-by-time interactions were detected regarding plasma VEGF concentrations (all *ps* > 0.05; Table 2). Despite significant reductions in MADRS anhedonia item scores at days 13 and 26 (all *p*s < 0.05; Figure 1), no significant changes in plasma VEGF concentrations were observed at day 13 when compared to baseline (*p* > 0.05) (Figure 1). No significant differences in plasma VEGF concentrations were found between antianhedonic responders and non-responders at days 13 and 26 (all *ps* > 0.05) (Figure 1).

**Table 2:** Comparison of MADRS anhedonia item scores and plasma VEGF concentrations between antianhedonic responders and non-responders in subjects suffering from TRD using linear mixed models.

**Variables**	**Group-by-time interaction**		**Time main effect**		**Group main effect**	
	**F**	***p-*value**	**F**	***p-*value**	**F**	***p-*value**
MADRS anhedonia item scores	55.2	**<0.001**	148.8	**<0.001**	72.6	**<0.001**
Plasma VEGF concentrations (ng/ml)	2.4	0.09	4.0	**0.02**	0.1	0.79

*Bolded values are p < 0.05. MADRS, the Montgomery-Åsberg Depression Rating Scale; VEGF, vascular endothelial growth factor; TRD, treatment-refractory depression*.

As shown in Table 3, correlation analysis of plasma VEGF concentrations and anhedonia symptoms as measured by the MADRS anhedonia item did not yield any significant relationships (all *ps* > 0.05).

**Table 3:** Relationship of baseline plasma VEGF concentrations and anhedonia symptoms in subjects suffering from TRD.

**Variables**		**MADRS anhedonia item scores**			**Change in MADRS anhedonia item scores**	
		**At baseline**	**At day 13**	**At day 26**	**At day 13**	**At day 26**
Baseline plasma VEGF concentrations (ng/ml)	*r*	−0.01	−0.13	−0.04	0.13	0.03
	*p*	0.94	0.24	0.74	0.23	0.77
Change in plasma VEGF concentrations at day 13 (ng/ml)	*r*	−0.06	−0.17	−0.24	0.13	0.19
	*p*	0.62	0.17	0.06	0.29	0.13
Change in plasma VEGF concentrations at day 26 (ng/ml)	*r*	−0.25	0.01	−0.11	−0.16	−0.04
	*p*	0.08	0.95	0.45	0.24	0.80

*MADRS, the Montgomery-Åsberg Depression Rating Scale; VEGF, vascular endothelial growth factor; r, Pearson coefficient of correlation; TRD, treatment-refractory depression*.

## Discussion

To our knowledge, this is the first report to determine whether plasma VEGF concentrations are involved in the rapid antianhedonic effects of ketamine. The major finding in the present study was that (1) consistent with previous studies (1, 2, 9, 10), ketamine exerted significant and rapid antianhedonic effects; (2) plasma VEGF concentrations showed no significant changes at day 13, and no significant difference in plasma VEGF concentrations was found in antianhedonic responders compared to non-responders at days 13 and 26; and (3) plasma VEGF concentrations showed no significant correlation with the observed antianhedonic effects in individuals treated with six ketamine infusions.

In this study, the observed rapid reduction in anhedonia symptoms after six ketamine infusions replicates findings from numerous earlier studies (1, 2, 9, 10). Of them, the Snaith–Hamilton Pleasure Scale (SHAPS) was used to evaluate the levels of anhedonia in some studies (1, 9, 10) but not all (2). In addition to the SHAPS, the Beck Depression Inventory (BDI) anhedonia item was used in Ballard et al. study (2). Similarly, a recent study examined the effects of esketamine on anhedonia symptoms by using MADRS item 8 (inability to feel) (30). In this study, the MADRS anhedonia item rather than a specific scale for anhedonia was used to evaluate anhedonia symptoms. Thus, a specific scale for anhedonia, such as the SHAPS and the Profile of Mood States (POMS), should be used to confirm these findings. Importantly, future studies should adopt a more specific assessment approach.

Preclinical trials have shown that rapid increases in VEGF in the medial prefrontal cortex (mPFC) are required for the behavioral action of ketamine (33). Neuronal VEGF–Flk-1 signaling in the mPFC was associated with the antidepressant actions of ketamine (19). VEGF also appeared to be critical for the behavioral effects of various antidepressants (20, 34, 35) and lamotrigine (36) in rodent models of depression. In a recent clinical study, a single infusion of ketamine increased the plasma mRNA levels of VEGF, supporting a role for VEGF in the action of ketamine (25). However, our data failed to demonstrate that plasma VEGF concentrations were significantly associated with ketamine's rapid antianhedonic effects in subjects with TRD. Similarly, a recent study also found that VEGF does not play a critical role in the observed antidepressant response to ketamine in depressed patients (24). However, the association of VEGF and ketamine's antisuicidal effects is unclear.

There were several limitations in the current study. First, since patient samples were limited to Chinese subjects suffering from treatment-refractory MDD or BD, the findings may not be fully generalizable. In addition, the pooling of individuals diagnosed with MDD and BD made the sample nonhomogeneous. Second, patients continued receiving psychotropic medication in this open-label real-world study, which may have affected the plasma VEGF concentrations and partly explained the contradictory findings between our study and early reports (25). Third, we did not directly measure brain VEGF levels since blood VEGF levels may not be related to brain VEGF concentrations (37). Fourth, other key neurobiological mediators of the ketamine response, such as phosphorylation of glycogen synthase kinase-3 (p-GSK-3) or mammalian target of rapamycin (mTOR) (38, 39), should be measured in future studies. Finally, the possible comorbid diagnosis such as a comorbidity of OCD or anxiety disorder was not reported in this study. Although treatment strategies for OCD, substance use disorders (SUD) and eating disorders (ED) are complex and difficult, ketamine and esketamine appeared to be effective in treating them (40).

## Conclusions

This preliminary study suggests that the antianhedonic effects of ketamine are not mediated by VEGF.

## Data Availability Statement

The original contributions presented in the study are included in the article/supplementary material, further inquiries can be directed to the corresponding authors.

## Ethics Statement

The studies involving human participants were reviewed and approved by the Affiliated Brain Hospital of Guangzhou Medical University. The patients/participants provided their written informed consent to participate in this study.

## Author Contributions

YPN: study design. WZ, YLZ, and CYW: data collection. WZ and LMG: analysis and interpretation of data. WZ: drafting of the manuscript. BZ, DFW, and YPN: critical revision of the manuscript. All the authors contributed to the final draft of the manuscript and approved the final version for publication.

## Funding

This study was funded by the National Natural Science Foundation of China (82101609), Scientific Research Project of Guangzhou Bureau of Education (202032762), Science and Technology Program Project of Guangzhou (202102020658), the Science and Technology Planning Project of Liwan District of Guangzhou (202004034), Guangzhou Health Science and Technology Project (20211A011045), Guangzhou science and Technology Project of traditional Chinese Medicine and Integrated Traditional Chinese and Western medicine (20212A011018), China International Medical Exchange Foundation (Z-2018-35-2002), Guangzhou Clinical Characteristic Technology Project (2019TS67), Science and Technology Program Project of Guangzhou (202102020658), and Guangdong Hospital Association (2019ZD06). The funders had no role in study design, data collection and analysis, decision to publish, or preparation of the manuscript.

## Conflict of Interest

The authors declare that the research was conducted in the absence of any commercial or financial relationships that could be construed as a potential conflict of interest.

## Publisher's Note

All claims expressed in this article are solely those of the authors and do not necessarily represent those of their affiliated organizations, or those of the publisher, the editors and the reviewers. Any product that may be evaluated in this article, or claim that may be made by its manufacturer, is not guaranteed or endorsed by the publisher.
